# Performance of the LIAISON PLEX yeast blood culture assay for identifying 16 invasive fungal pathogens in blood cultures

**DOI:** 10.1128/jcm.00362-25

**Published:** 2025-06-02

**Authors:** Christopher L. Emery, Neelam Dhiman, Siu-Kei Chow, Gwyn Peterson, Paul Granato, Brian Bernier, Janet Farhang

**Affiliations:** 1 Indiana University Health 22529, Indianapolis, Indiana, USA; 2 Quest Diagnostics, Lewisville, Texas, USA; 3 MultiCare Health System 14458, Tacoma, Washington, USA; 4 Laboratory Alliance of Central New York 51972, Liverpool, New York, USA; 5 Luminex Corporation 17737 , Austin, Texas, USA; University of Calgary, Calgary, Alberta, Canada

**Keywords:** *Candida*, *Cryptococcus*, blood culture, *in vitro* diagnostic assay, molecular syndromic panel, sample-to-answer

## Abstract

Fungi frequently cause potentially life-threatening bloodstream infections, particularly in immunocompromised and hospitalized individuals. Molecular methods can allow earlier pathogen identification for faster optimization of appropriate therapy. This multisite study evaluated the analytical and clinical performance of the automated multiplex LIAISON PLEX Yeast Blood Culture nucleic acid assay (Luminex Corporation, Northbrook, IL), which detects and identifies 14 *Candida* and 2 *Cryptococcus* pathogens from positive blood cultures. Samples included 69 prospectively collected specimens, 63 pre-selected samples, and 829 contrived specimens. The assay demonstrated 100% positivity in detecting target fungal pathogens and 0% positivity in negative blood cultures. All 13 tested bottle/matrix types gave 100% detection of six evaluated pathogens. Limits of detection ranged from 7.77 × 10^2^ CFU/mL (*Candida famata*) to 2.83 × 10^5^ CFU/mL (*Candida albicans*). The assay identified five strains tested for each of the 16 target species, indicating good inclusivity. No cross-reactivity occurred with 40 bacterial strains. Cross-reactivity with 2 of 37 off-target fungi (*Candida* [*Yarrowia*] *deformans* and *Candida pseudohaemulonii*) was predicted by *in silico* sequence analyses. Low concentrations of on-panel fungi were detectable alongside high concentrations of potential fungal and bacterial confounders. Reproducibility was 100% within-lab and 99.7% across sites. For combined prospective plus pre-selected specimens, sensitivity/positive percent agreement (PPA) was 100% for detected fungal pathogens, and specificity/negative percent agreement (NPA) was 97.6%‒100.0%. Common pathogens in prospective samples were *Candida glabrata* (36%) and *C. albicans* (25%). Contrived specimen PPA was 100%, and NPA was ≥99.6%. The LIAISON PLEX Yeast Blood Culture assay provides outstanding sensitivity and specificity for rapidly identifying and differentiating fungal pathogens in positive blood culture specimens.

**IMPORTANCE:**

Bloodstream fungal infections have serious risks of illness and death. Optimal treatment requires early pathogen identification so appropriate antifungal therapy can be immediately started. Blood culture is standard for confirming fungal infection but takes several days for results and may not provide detailed pathogen information. The LIAISON PLEX Yeast Blood Culture assay quickly analyzes fungus-positive blood cultures to identify 16 pathogenic fungi, including multiple *Candida* spp. that are common causes of bloodstream infection. This study demonstrated a high degree of test accuracy and reliability in rapidly identifying the infective pathogen in blood cultures from 132 patients with demonstrated culture-positive fungal infections. Assay performance was confirmed in 829 contrived samples, whereby samples were spiked with different fungal pathogens.

## INTRODUCTION

Systemic fungal infections account for 3.8 million deaths per year worldwide, with approximately 68% of fatalities directly attributed to the infection (1). Many *Candida* spp. are normal residents of the human skin, gastrointestinal, and genitourinary microbiomes. Infection occurs when host defenses are suppressed (e.g., HIV infection, organ transplant patients, and chemotherapy for malignancy) or when resident microbiome populations become imbalanced and permit *Candida* overgrowth (e.g., from broad-spectrum antibiotic use). Invasive candidiasis is composed of bloodstream infections (candidemia) and hematogenous infection of deep tissues (e.g., heart, abdominal cavity, and central nervous system) that is often accompanied by candidemia (2). The annual global incidence of invasive candidiasis is 700,000 cases (3), with a fatality rate of approximately 40% (4). In the USA, hospitalization for fungal infections costs an estimated $4.6 billion in 2017; of this, *Candida* spp. alone accounted for $1.4 billion (5).

Of the different *Candida* spp. known to cause infection in humans, the most common species responsible for more than 90% of invasive infections are *Candida albicans*, *Candida glabrata* (*Nakaseomyces glabratus*), *Candida parapsilosis*, *Candida tropicalis*, and *Candida krusei* (*Pichia kudriavzevii*) (6). While *C. albicans* remains the most common species causing infections, there has been an increase in candidemia due to non-*albicans Candida* spp. Notably, *Candida auris* (*Candidozyma auris*), only identified in 2009 by Japanese researchers, has emerged as a novel multidrug-resistant pathogen with high outbreak potential (7). The World Health Organization (WHO) recently compiled a fungal priority pathogen list (WHO FPPL) to rank 19 common and emerging fungal pathogens based on public health threat, antifungal resistance, global distribution, and epidemiological trends (8). Four of the 19 pathogens evaluated are categorized as of “critical priority” for healthcare and research and development efforts: *C*. *albicans*, *C. auris*, *Cryptococcus neoformans*, and *Aspergillus fumigatus*. Of the eight fungal pathogens deemed high priority, three are *Candida* spp.

Diagnosis of invasive fungal disease remains challenging because of the overlap of clinical features with bacterial infections, reliance on blood culture assays with limited sensitivity that can take several days to yield positive results, and the often invasive nature of sampling required for histopathological evaluation (9). Standard blood culture has a sensitivity of 21%‒71% in identifying candidemia in autopsy-proven samples, trending lower in early disease and in patients who have begun prophylactic antifungal therapy (10). Blood culturing for *Candida* spp. typically has 72‒96 h turnaround times (longer for some species), which delays treatment initiation and leads to increased mortality (6). Initially, positive blood culture results often require detailed follow-up testing to confirm pathogen identity and characterize its resistance to different antifungal drug classes in the current medical armament (11). Alternative diagnostic approaches such as matrix-assisted laser desorption/ionization time-of-flight mass spectrometry and non-culture techniques such as PCR analysis and beta-D-glucan testing may improve *Candida* detection (12–14).

The current study evaluated the analytical and clinical performance of a qualitative multiplexed molecular *in vitro* diagnostic test, the LIAISON PLEX Yeast Blood Culture (BCY) Assay (Luminex Corporation). Requiring only approximately 1 minute of hands-on time and 2 h from sample to result, this automated BCY Assay simultaneously evaluates positive blood culture samples for the presence of fungal nucleic acids to identify 14 pathogenic *Candida* spp. and 2 *Cryptococcus* spp. that cause most cases of systemic fungal infection (1).

## MATERIALS AND METHODS

### LIAISON PLEX Yeast Blood Culture Assay

The Food and Drug Administration (FDA)-cleared LIAISON PLEX BCY Assay (FDA Clearance # K240627) is a qualitative multiplex nucleic acid-based diagnostic assay that detects 14 targets for the identification of 16 fungal pathogens commonly responsible for bloodstream infection (15). The targets included in the LIAISON PLEX BCY Assay are *Candida albicans*, *Candida auris* (*Candidozyma auris*), *Candida dubliniensis*, *Candida famata* (*Debaryomyces hansenii*), *Candida glabrata* (*Nakaseomyces glabratus*), *Candida guilliermondii* (*Meyerozyma guilliermondii*), *Candida kefyr* (*Kluyveromyces marxianus*), *Candida krusei* (*Pichia kudriavzevii*), *Candida lipolytica* (*Yarrowia lipolytica*), *Candida lusitaniae* (*Clavispora lusitaniae*), *Candida parapsilosis*, *Candida tropicalis*, *Candida haemulonii*/*Candida duobushaemulonii* (combined target), and *Cryptococcus neoformans*/*Cryptococcus gattii* (combined target). The BCY Assay is intended to confirm and extend findings from blood cultures that Gram stain positive for yeast, reliably and more quickly than subsequent culture-based downstream approaches. The LIAISON PLEX System automates sample analysis in a fully integrated system that performs all of the assay steps in a single cartridge (16). Briefly, broth samples from blood culture with Gram stain indicating yeast are vortexed, and 300 µL volumes are pipetted into the assay cartridge; the Sample ID barcode and cartridge ID barcode are scanned using the system’s handheld scanner; the sample cartridge is loaded into the instrument, and run parameters are specified and initiated. Sample preparation and loading require approximately 1 minute of hands-on time, and results are available in about 2 h.

Automated LIAISON PLEX sample processing involves magnetic bead-based nucleic acid isolation and subsequent amplification by multiplex PCR to generate target-specific amplicons that are then hybridized to specific capture oligonucleotide probes microarrayed on a glass slide. The bound target DNA amplicons are subsequently decorated by hybridizing gold nanoparticle-conjugated target-specific oligonucleotide probes, followed by colloidal silver signal enhancement. The instrument then assesses light scatter by the silver-enhanced microarray spots to characterize the presence (detected) or absence (not detected) of each fungal analyte.

### Clinical performance

Diagnostic accuracy of the LIAISON PLEX BCY Assay was evaluated using 70 specimens prospectively collected between June 2023 and October 2023 from four geographically diverse clinical sites across the USA. Samples were remnant, de-identified, blood culture specimens from patients exhibiting clinical signs and symptoms of bloodstream infection, evidenced by positive identification with a continuous monitoring blood culture system. All prospectively collected specimen identities were determined by the standard of care culture and mass spectrometry (matrix-assisted laser desorption ionization time-of-flight [MALDI-TOF]) methods used at each clinical site. Continuous monitoring blood culture systems included the BacT/ALERT VIRTUO (bioMérieux SA, Marcy-l'Étoile, France), BacT/ALERT 3D (bioMérieux SA), and BD BACTEC (Becton, Dickinson and Company; Franklin Lakes, NJ).

Clinical runs and reruns were performed on the LIAISON PLEX System by trained operators at four clinical sites. Because several fungal pathogen targets exhibited low prevalence rates in the prospective study, the prospective sample set was supplemented with 63 pre-selected de-identified blood culture specimens obtained from six vendors in the USA (University of Texas Medical Branch, Galveston, TX; NorthShore University Health System, Evanston, IL; Southwest Bio, Albuquerque, NM; Laboratory Alliance of Central New York, Liverpool, NY; Kaleida Health, Buffalo, NY; and Discovery Life Sciences, Huntsville, AL). Pre-selected specimen collection dates spanned December 2019‒August 2023. Pre-selected specimens were identified by standard of care culture methods and confirmed by the BIOFIRE Blood Culture Identification 2 (BCID2) panel prior to study enrollment and were stored at −70°C before testing. Pre-selected specimens were tested in a randomized, blinded manner with negative specimens at one site in October 2023.

Contrived specimens were tested to supplement the positive clinical specimens in the prospective and pre-selected study cohorts for all targets. Contrived specimens were prepared from multiple strains of each organism obtained from commercial sources. Each strain was subcultured to Sabouraud dextrose agar and incubated 1-3 days until visible colony growth was achieved. Isolated colonies were suspended in phosphate-buffered saline to a concentration of approximately 3.0 × 10^8^ CFU/mL (1.0 McFarland standard). A 100 µL aliquot of the suspension was used to inoculate BACT/ALERT FA Plus blood culture bottles (bioMérieux) containing whole blood from individual healthy donors that were purchased from a commercial source. Each inoculated blood culture bottle was grown to positivity on a BACT/ALERT 3D continuous monitoring automated blood culture system (bioMérieux), and contents were collected within 8 h of bottle positivity. A minimum of 50 contrived specimens were prepared for each target that did not reach 30 positive specimens in the combined prospective and pre-selected arms of the study. A total of 829 specimens were contrived, blinded, randomized, and tested along with negative specimens at four testing sites during June 2023–August 2023.

### Analytical performance overview

The LIAISON PLEX BCY Assay was evaluated for limit of detection (LoD), compatibility with diverse commercial culture bottles/matrices, detectability of multiple target variants, potential interfering substances, impact of diverse fungal and bacterial co-infections as potential interferents, run-to-run carryover, and intralab/inter-operator repeatability and site-to-site reproducibility. Reference fungal cultures were from the American Type Culture Collection (ATCC, Manassas, VA), Fungal Biodiversity Centre (CBS, Utrecht, Netherlands), and the US Biodefense and Emerging Infections Research Resources Repository (BEI Resources; National Institute of Allergy and Infectious Diseases, Bethesda, MD).

### Target detection

Fourteen organisms representing each of the 14 reportable targets included in the LIAISON PLEX BCY Assay were grown in blood culture media bottles and evaluated by the assay at bottle positivity (average concentrations ranging from 4.37 × 10^5^ to 3.61 × 10^7^ CFU/mL) and after an additional 8 h incubation (average concentrations ranging from 4.10 × 10^6^ to 1.47 × 10^8^ CFU/mL). Three bottles at each incubation time were grown for each organism and tested in triplicate for a total of nine replicates per organism. Average concentrations for each organism at each incubation time are shown in Table S1. A negative blood matrix specimen served as control.

### Matrix equivalency

We evaluated the impact of 12 different bottle matrices from three manufacturers on assay performance at clinically relevant organism concentrations. Each matrix was tested with negative blood matrix, two positive control panels containing a total of six pathogenic fungi (*C. albicans*, *C. tropicalis*, *C. neoformans*, *C. glabrata*, *C. guilliermondii*, and *C. kefyr*), and five off-panel bacterial strains (*Acinetobacter baumannii*, *Escherichia coli*, *Klebsiella pneumoniae*, *Pseudomonas aeruginosa*, and *Staphylococcus aureus*). The concentrations of organisms in the fungal and bacterial panels were 4.4 × 10^5^ CFU/mL for *C. albicans*, 1.2 × 10^7^ CFU/mL for *C. tropicalis*, 1.84 × 10^6^ CFU/mL for *C. neoformans*, 3.8 × 10^6^ CFU/mL for *C. glabrata*, 1.86 × 10^7^ CFU/mL for *C. guilliermondii*, 6.3 × 10^6^ CFU/mL for *C. kefyr*, and 1 × 10^8^ CFU/mL for all of the bacteria. The compatible blood culture bottle matrices are listed in Table S2.

### Limit of detection

An LoD study was performed to determine the analytical sensitivity of the LIAISON PLEX BCY Assay. Two strains of each organism corresponding to a single species target and one strain of each organism corresponding to a combined species target included in the assay were tested (28 total strains). Twenty replicates were tested per strain, and the LoD was defined as the lowest concentration at which ≥95% (≥19 out of 20) of samples tested positive.

### Analytical reactivity (inclusivity)

Analytical reactivity of the LIAISON PLEX BCY Assay was assessed using 80 total strains, 5 strains for each of the 16 yeast species included in the assay. Each strain was prepared to a concentration representative of a positive blood culture bottle and tested in triplicate. The prepared concentrations were the same for each strain tested within a species, and the concentrations tested for each of the 16 yeast species ranged from 4.4 × 10^5^ CFU/mL for *C. albicans* to 2.2 × 10^7^ CFU/mL for *C. lusitaniae*.

### Analytical specificity (cross-reactivity)

Analytical cross-reactivity was evaluated by using the LIAISON PLEX BCY Assay to attempt detection of off-panel and potentially cross-reactive organisms, including those phylogenetically related to the on-panel organisms and organisms likely to be present in blood culture samples. Forty off-panel bacteria and 37 off-panel yeast species were tested at clinically relevant concentrations. Bacteria were tested at concentrations of 1 × 10^8^ CFU/mL, with the exception of *Micrococcus luteus* (8.9 × 10^6^ CFU/mL) and *Stenotrophomonas maltophilia* (8.9 × 10^7^ CFU/mL), which were tested at the concentration achieved by culture. Yeasts were tested at concentrations of 1 × 10^7^ CFU/mL, with the exception of *Talaromyces marneffei* (ATCC 18224), which was provided at a concentration of 1.45 × 10^6^ spores/mL. See Table S3 for a list of bacterial and fungal organisms tested.

### Interfering substances

An interfering substance study evaluated potential effects of non-microbial assay interference by 18 substances consisting of 5 endogenous substances (unconjugated bilirubin, conjugated bilirubin, hemoglobin, intralipid/triglycerides, and γ-globulin) and 13 exogenous substances (sodium polyanethol sulfonate, amoxicillin clavulanate, amphotericin B, caspofungin, ceftriaxone, ciprofloxacin, fluconazole, flucytosine, gentamicin sulfate, heparin, imipenem, tetracycline, and vancomycin), which may be present during collection or within clinical blood samples. Two positive panels containing three on-panel organisms each (*C. albicans*, *C. tropicalis*, and *C. neoformans* and *C. glabrata*, *C. guilliermondii*, and *C. kefyr*) and negative blood matrix were used to test each interfering substance at clinically relevant concentrations. Interfering substances and concentrations tested are shown in Table S4.

### Carry-over/cross-contamination

High-positive *C. lusitaniae* samples (ring-positive plus an additional 8 h incubation of blood culture) and negative samples (negative blood matrix) were assayed in an alternating series by two operators. Immediately following the testing of a positive sample, a negative sample was run in the same bay to evaluate carry-over contamination. This alternating pattern was continued over five consecutive runs across two LIAISON PLEX instruments for a total of 30 positive and 30 negative tests.

### Competitive inhibition/co-infection and microbial interference

The impact of high-concentration on-panel fungal co-infection and high-concentration off-panel bacterial co-infection on low-concentration on-panel fungal target detection was evaluated. Representative organisms were grown to blood culture bottle positivity (*C. albicans* at 4.4 × 10^5^ CFU/mL, *C. glabrata* at 3.8 × 10^6^ CFU/mL, and *C. parapsilosis* at 1.5 × 10^7^ CFU/mL) and were tested in pair-wise combinations with three highly concentrated (ring positive plus an additional 8 h incubation) on-panel fungi (*C. albicans* at 1.7 × 10^7^ CFU/mL, *C. glabrata* at 1.6 × 10^8^ CFU/mL, and *C. parapsilosis* at 5.5 × 10^7^ CFU/mL) and nine highly concentrated (all at 1 × 10^8^ CFU/mL) potentially interfering bacteria. The 33 pairwise combinations were tested in triplicate for a total of 99 replicates.

### Multisite reproducibility

Site-to-site reproducibility of the LIAISON PLEX BCY Assay was evaluated by testing one lot of BCY Assay cartridges by two operators at each of three testing sites. Four samples included (i) negative blood matrix; (ii) contrived negative sample containing an off-panel bacteria (*E. coli* at 1 × 10^8^ CFU/mL) and a positive target panel containing three on-panel targets (*C. albicans*, *C. tropicalis*, and *C. neoformans*) prepared to (iii) ring-positive (4.3 × 10^5^ CFU/mL for *C. albicans*, 1.2 × 10^7^ CFU/mL for *C. tropicalis*, and 1.84 × 10^6^ CFU/mL for *C. neoformans*); and (iv) ring-positive plus 8 h additional incubation (1.54 × 10^7^ CFU/mL for *C. albicans*, 3.9 × 10^7^ CFU/mL for *C. tropicalis*, and 5.5 × 10^6^ CFU/mL for *C. neoformans*) concentrations. Samples were randomized and blinded to the operators, and each operator tested each sample concentration in triplicate on each of five non-consecutive testing days.

### Within-laboratory precision/repeatability

Inter-operator precision at a single site was evaluated across two operators, two LIAISON PLEX Systems, and one lot of BCY Assay cartridges. Four samples included (i) negative blood matrix; (ii) contrived negative sample containing an off-panel bacteria (*E. coli*) and a positive target panel containing three on-panel targets (*C. albicans*, *C. tropicalis*, and *C. neoformans*) prepared to (iii) ring positive (4.3 × 10^5^ CFU/mL for *C. albicans*, 1.2 × 10^7^ CFU/mL for *C. tropicalis*, and 1.84 × 10^6^ CFU/mL for *C. neoformans*) and (iv) ring-positive plus 8 h additional incubation (1.54 × 10^7^ CFU/mL for *C. albicans*, 3.9 × 10^7^ CFU/mL for *C. tropicalis*, and 5.5 × 10^6^ CFU/mL for *C. neoformans*) concentrations. Samples were randomized and blinded to the operators, and each operator tested each sample concentration in triplicate on each of five non-consecutive testing days.

### Statistical analysis

Data are presented as *n* (%) or value ± 95% confidence intervals (CIs), as indicated. Statistical software included SAS v.9.4 (SAS Corp, Cary, NC).

## RESULTS

### Clinical trial subject and specimen details

Demographic and sample details for included prospective and pre-selected specimens are shown in Table 1. Of 70 prospectively enrolled specimens, one was disqualified from analysis due to an inconclusive Gram stain result. No specimen in the pre-selected cohort was disqualified for any reason. Of the 837 samples enrolled in the contrived cohort, 8 (0.96%) were excluded from analysis due to incorrectly labeled stock material.

**TABLE 1:** Demographics and sampling details

Parameter	Prospective (*N* = 69)*^a^*	Pre-selected (*N* = 63)
	Specimens, *n* (%)	Specimens, *n* (%)
Gender		
Male	36 (52.2)	24 (38.1)
Female	32 (46.4)	25 (39.7)
Gender Unknown	1 (1.4)	14 (22.2)
Total	69 (100.0)	63 (100.0)
Age (years)		
0–1	3 (4.3)	0 (0.0)
>1–5	0 (0.0)	1 (1.6)
>5–21	1 (1.4)	0 (0.0)
>21–65	41 (59.4)	27 (42.9)
>65	23 (33.3)	19 (30.2)
Age unknown	1 (1.4)	16 (25.4)
Total	69 (100.0)	63 (100.0)
Subject status		
Emergency room	5 (7.2)	0 (0.0)
Hospitalized	22 (31.9)	2 (3.2)
Status unknown	42 (60.9)	61 (96.8)
Total	69 (100.0)	63 (100.0)
Blood culture bottle type		
BD BACTEC lytic anaerobic	3 (4.3)	2 (3.2)
BD BACTEC Plus aerobic	14 (20.3)	17 (27.0)
BD BACTEC standard aerobic	5 (7.2)	29 (46.0)
BacT/ALERT FA Plus	40 (58.0)	0 (0.0)
BacT/ALERT FN Plus	1 (1.4)	0 (0.0)
BacT/ALERT PF Plus	2 (2.9)	0 (0.0)
BacT/ALERT SA standard aerobic	3 (4.3)	0 (0.0)
BacT/ALERT SN standard anaerobic	1 (1.4)	0 (0.0)
Bottle type unknown	0 (0.0)	15 (23.8)
Total	69 (100.0)	63 (100.0)

^*a*^ One of 70 prospectively collected specimens was disqualified and removed from analysis due to not meeting inclusion criteria (i.e., had inconclusive Gram stain results) after enrollment. No pre-selected samples were disqualified after enrollment.

### Clinical performance

The clinical performance of the LIAISON PLEX BCY Assay was compared to the standard of care culture-based/MALDI-TOF identification methods at the clinical sites for all targets. Of the 132 prospective (*n* = 69) and pre-selected (*n* = 63) specimens included in the study analysis, 130 (98.5%) generated valid results (i.e., detected or not detected) on the first attempt. Two samples generated invalid results on the first attempt, and both gave no call due to internal control failure. Of the two samples (1.5%) with initially invalid results, both produced valid assay results after a single retest. Allowing a single retest of any initially invalid results, all 132 specimens yielded valid BCY Assay results for a final success rate of 100%. Cumulative clinical performance data (sensitivity/positive percent agreement [PPA] and specificity/negative percent agreement [NPA], with 95% CIs) of the LIAISON PLEX BCY Assay versus the reference method are shown in Table 2 for combined prospective plus pre-selected specimens. Sensitivity/PPA was 100% for all detected fungal pathogens, and specificity/NPA was 97.6%‒100% for all pathogens. Three of five samples positive by the LIAISON PLEX BCY Assay but negative by the reference method were confirmed positive by the BCID2 molecular assay, indicating a true detection. Of the 132 prospective and pre-selected samples, 42 were cultured on the BACT/ALERT VIRTUO instrument with BACT/ALERT FA Plus media (bioMérieux, Durham, NC), 61 on BD BACTEC with BD BACTEC Lytic Anaerobic and BD BACTEC Plus Aerobic media (Becton, Dickinson and Company), and five on BACT/ALERT 3D with BACT/ALERT SA Standard Aerobic, BACT/ALERT SN Standard Anaerobic, and BACT/ALERT PF Plus.

**TABLE 2:** Clinical performance of the LIAISON PLEX BCY Assay with combined prospective plus pre-selected specimens^*f*^

Fungal pathogen	Sensitivity/PPA*^a^*			Specificity/NPA*^b^*		
	TP/(TP + FN)	Sensitivity/PPA (%)	95% CI (%)	TN/(TN + FP)	Specificity/NPA (%)	95% CI (%)
*Candida albicans*	34/34	100	89.8–100.0	97/98^*c*^	99.0	94.4–99.8
*Candida auris*	4/4	100	51.0–100.0	128/128	100	97.1–100.0
*Candida dubliniensis*	0/0	NA	NA	132/132	100	97.2–100.0
*Candida famata*	0/0	NA	NA	132/132	100	97.2–100.0
*Candida glabrata*	46/46	100	92.3–100.0	86/86	100	95.7–100.0
*Candida guilliermondii*	0/0	NA	NA	132/132	100	97.2–100.0
*Candida haemulonii*/*C. duobushaemulonii*	0/0	NA	NA	132/132	100	97.2–100.0
*Candida kefyr*	1/1	100	20.7–100.0	131/131	100	97.2–100.0
*Candida krusei*	4/4	100	51.0–100.0	128/128	100	97.1–100.0
*Candida lipolytica*	0/0	NA	NA	132/132	100	97.2–100.0
*Candida lusitaniae*	2/2	100	34.2–100.0	130/130	100	97.1–100.0
*Candida parapsilosis*	17/17	100	81.6–100.0	114/115^*d*^	99.1	95.2–99.8
*Candida tropicalis*	6/6	100	61.0–100.0	123/126^*e*^	97.6	93.2–99.2
*Cryptococcus neoformans*/*C. gattii*	5/5	100	56.6–100.0	127/127	100	97.1–100.0

^*a*^ Sensitivity is designated for prospective sample testing, while PPA is designated for pre-selected sample testing.

^*b*^ Specificity is designated for prospective sample testing, while NPA is designated for pre-selected sample testing.

^*c*^ The one *Candida albicans* false positive was positive by the BCID2 molecular assay.

^*d*^ The one *Candida parapsilosis* false positive was positive by the BCID2 molecular assay.

^*e*^ One of three *Candida tropicalis* false positives was positive by the BCID2 molecular assay.

^*f*^ FP, false positive; NA, not available; TN, true negative; TP, true positive.

The top-three most commonly detected fungal pathogens in the prospectively collected US sample set were *C. glabrata* at 36% (*n* = 25/69), *C. albicans* at 25% (*n* = 17/69), and *C. parapsilosis* at 16% (*n* = 11/69); collectively accounting for 77% of identified infections. *C. auris* was identified in 6% (*n* = 4/69) of prospectively collected specimens, and *Cryptococcus neoformans*/*C. gattii* was not identified in this cohort. Assay performance was nearly indistinguishable between prospective versus pre-selected specimens, for all fungal pathogen targets (Table S5).

Of the 829 specimens included in the contrived study analysis, 803 specimens (96.9%) generated valid LIAISON PLEX BCY Assay results (i.e., detected or not detected) on the first attempt (Table 3). For the 26 specimens that were initially invalid, 17 were due to aborted runs caused by instrument issues, and 9 had no call due to internal control failure. Of the 26 specimens retested (3.1%), 24 specimens generated a valid result after a single retest for a final success rate of 99.7% (827 out of 829). The PPA was 100% for all fungal pathogens, and the NPA was 99.6%‒100.0%.

**TABLE 3:** Clinical performance of the LIAISON PLEX BCY Assay with contrived specimens

Pathogen target	Positive percent agreement			Negative percent agreement		
Analyte	TP/(TP + FN)	PPA (%)	95% CI (%)	TN/(TN + FP)	NPA (%)	95% CI (%)
*Candida albicans*	50/50	100	92.9–100.0	777/777	100.0	99.5–100.0
*Candida auris*	50/50	100	92.9–100.0	777/777	100.0	99.5–100.0
*Candida dubliniensis*	50/50	100	92.9–100.0	777/777	100.0	99.5–100.0
*Candida famata*	50/50	100	92.9–100.0	776/777^*a*^	99.9	.099.3–100.0
*Candida glabrata*	49/49	100	92.7–100.0	778/778	100.0	99.5–100.0
*Candida guilliermondii*	50/50	100	92.9–100.0	777/777	100.0	99.5–100.0
*Candida haemulonii*/*C. duobushaemulonii*	50/50	100	92.9–100.0	777/777	100.0	99.5–100.0
*Candida kefyr*	50/50	100	92.9–100.0	777/777	100.0	99.5–100.0
*Candida krusei*	50/50	100	92.9–100.0	777/777	100.0	99.5–100.0
*Candida lipolytica*	50/50	100	92.9–100.0	777/777	100.0	99.5–100.0
*Candida lusitaniae*	50/50	100	92.9–100.0	777/777	100.0	99.5–100.0
*Candida parapsilosis*	50/50	100	92.9–100.0	776/777*^b^*	99.9	99.3–99.9
*Candida tropicalis*	49/49	100	92.7–100.0	775/778^*c*^	99.6	98.9–99.9
*Cryptococcus neoformans*/*Cryptococcus gattii*	100/100	100	96.3–100.0	727/727	100.0	99.5–100.0

^*a*^ The one *Candida famata* false positive was not further tested.

^*b*^ The one *Candida parapsilosis* false positive was negative by the BCID2 molecular assay.

^*c*^ The three *Candida tropicalis* false positives were negative by the BCID2 molecular assay.

Multiple organisms were detected in six prospective specimens. All six co-infections contained two organisms. Of these, one was concordant with the reference method, and five contained one organism that was not detected by the reference method, but three of these were detected by the BCID2 molecular assay (Table 4).

**TABLE 4:** Co-infections detected by LIAISON PLEX BCY Assay in prospective specimens

Co-infection detected	False positive target
*Candida albicans*/*Candida parapsilosis*	*Candida parapsilosis^a^*
*Candida albicans*/*Candida tropicalis*	*Candida albicans^b^*
*Candida auris*/*Candida tropicalis*	*Candida tropicalis*
*Candida glabrata*/*Candida tropicalis*	*Candida tropicalis* (*n* = 2)*^c^*
*Candida kefyr*/*Candida tropicalis*	NA^*d*^

^*a*^ The one *Candida parapsilosis* false positive was positive by the BCID2 molecular assay.

^*b*^ The one *Candida albicans* false positive was negative by the BCID2 molecular assay.

^*c*^ One of two *Candida tropicalis* false positives was positive by the BCID2 molecular assay.

^*d*^ NA, not available.

One prospective sample contained *Candida orthopsilosis* as identified by the standard of care testing methods but was negative on the LIAISON PLEX BCY Assay as this organism was not included on the panel.

### Target detectability

The LIAISON PLEX BCY Assay demonstrated 100% positivity in detecting 14 fungal pathogen target-positive samples and 0% positivity when tested with negative blood (Table S1). Average fungal concentration at ring positive ranged from 4.37 × 10^5^ CFU/mL for *C. albicans* to 3.61 × 10^7^ CFU/mL for *C. lusitaniae*, and ring positive plus 8 h additional incubation ranged from 4.10 × 10^6^ CFU/mL for *C. lipolytica* to 1.47 × 10^8^ CFU/mL for *C. glabrata*. The average concentrations for each target across the three replicate blood cultures at ring positive and ring positive plus 8 h additional incubation are shown in Table S1.

### Matrix equivalency

A matrix equivalency study was conducted to evaluate 12 different blood culture bottle matrices. All bottles demonstrated 100% detection of six fungal pathogens (*C. albicans*, *C. tropicalis*, *C. neoformans*, *C. glabrata*, *C. guilliermondii*, and *C. kefyr*), with 0% false detection of five bacterial strains (*A. baumannii*, *E. coli*, *K. pneumoniae*, *P. aeruginosa*, and *S. aureus*) or negative blood cultures. Additionally, the BioMérieux BACT/Alert FA Plus was used during the analytical testing of this assay and was also considered compatible. Thus, 13 bottle types tested provided expected results and were deemed compatible with the LIAISON PLEX Yeast BCY (Table S2).

### Limit of detection

The LoD for targets ranged from a low concentration (highest sensitivity) of 7.77 × 10^2^ CFU/mL for one *Candida famata* variant (ATCC 20278) to a high concentration of 2.83 × 10^5^ CFU/mL for one *Candida albicans* variant (ATCC 14053) (Table S6).

### Analytical reactivity (inclusivity)

The LIAISON PLEX BCY Assay identified all 80 fungal strains tested, which included 5 strains for each of the 16 yeast species included in the assay, indicating a high degree of inclusivity (data not shown).

### Analytical specificity (cross-reactivity)

None of the 40 bacterial species cross-reacted with any fungal target in the BCY Assay (Table S3). Of the 37 off-panel fungi tested, none cross-reacted with any fungal target in the BCY Assay with the exception of *Candida* (*Yarrowia*) *deformans*, which had 100% detection for the BCY target, *Candida lipolytica*, when tested at concentrations greater than 1.0 × 10^4^ CFU/mL, and *Candida pseudohaemulonii*, which had 100% detection for the BCY target, *Candida haemulonii*/*C. duobushaemulonii*, when tested at concentrations greater than 1.0 × 10^2^ CFU/mL (Table S3). These cross-reacting targets were predicted by *in silico* analysis of the LIAISON PLEX BCY Assay oligo designs (data not shown).

### Competitive inhibition/co-infection and microbial interference

Low concentrations (ring-positive) of three on-panel fungal targets were detected 100% of the time when co-assayed in the presence of high concentrations of any of three different on-panel fungal targets (*n* = 18 cumulative determinations, Table 5) and 100% of the time in the presence of high concentrations of any of nine potential bacterial interferents (*n* = 81 cumulative determinations, Table S7).

**TABLE 5:** Lack of inhibition of LIAISON PLEX BCY Assay target detection by high concentrations of other fungal organisms

On-panel high-concentration target	High-concentration target positivity (%)	On-panel low-concentration target	Low-concentration target positivity
*Candida parapsilosis* (5.5 × 10^7^ CFU/mL)	100 (3 out of 3)	*Candida albicans* (4.4 × 10^5^ CFU/mL)	100 (3 out of 3)
*Candida parapsilosis* (5.5 × 10^7^ CFU/mL)	100 (3 out of 3)	*Candida glabrata* (3.8 × 10^6^ CFU/mL)	100 (3 out of 3)
*Candida glabrata* (1.6 × 10^8^ CFU/mL)	100 (3 of 3)	*Candida parapsilosis* (1.5 × 10^7^ CFU/mL)	100 (3 out of 3)
*Candida glabrata* (1.6 × 10^8^ CFU/mL)	100 (3 of 3)	*Candida albicans* (4.4 × 10^5^ CFU/mL)	100 (3 out of 3)
*Candida albicans* (1.5 × 10^7^ CFU/mL)	100 (3 of 3)	*Candida glabrata* (3.8 × 10^6^ CFU/mL)	100 (3 out of 3)
*Candida albicans* (1.5 × 10^7^ CFU/mL)	100 (3/3)	*Candida parapsilosis* (1.5 × 10^7^ CFU/mL)	100 (3 out of 3)

### Interfering substances

Six on-panel fungal targets (*C. albicans*, *C. tropicalis*, *C. neoformans*, *C. glabrata*, *C. guilliermondii*, and *C. kefyr*) were used to evaluate the impact of 18 potentially interfering substances, including 5 endogenous substances and 13 exogenous substances (as shown in Table S4), that could be present in clinical blood samples. At the concentrations tested (Table S4), none of the 18 potentially interfering substances (0.0%) interfered with the performance of the LIAISON PLEX BCY. However, fluconazole showed interference when it was tested at a concentration of 25 mg/L and resulted in a false-negative result for *C. albicans*.

### Multisite reproducibility

Overall percent agreement across a total of six operators at three sites was 99.7% for both positive and negative samples when tested by the LIAISON PLEX BCY Assay (Table S8). The sole incongruity was a single *C. albicans* sample at 4.3 × 10^5^ CFU/mL that was missed (false negative) by one operator at one site.

## DISCUSSION

This study demonstrated the outstanding analytical and clinical performance of the LIAISON PLEX Yeast Blood Culture assay for detection and identification of 16 yeast species in positive blood cultures. Quick and accurate identification of the specific pathogen(s) responsible for bloodstream infection is essential for timely initiation of the most appropriate antimicrobial therapeutic regimen. The LIAISON PLEX BCY Assay quickly and non-invasively confirms and expands fungal pathogen information from initially positive blood cultures, which may improve patient outcomes by informing the timely selection of the most appropriate medical treatment.

Analytical performance of the LIAISON PLEX BCY Assay was excellent, as demonstrated by the reliable detection of multiple strains of each fungal target (i.e., high inclusivity), compatibility with 13 commonly employed culture bottle/matrix combinations, minimal cross-reactivity with off-panel organisms, and the ability to detect on-panel organisms in the presence of high concentrations of potentially interfering or competing microorganisms. In analytical assessments, 97% of specimens generated valid results on the first attempt; after a single retest of inconclusive samples using a new assay cartridge, the final success rate was 99.7%. Inter-site and inter-operator reproducibility were consistently 100%, reflecting the technical simplicity of the streamlined multiplex assay system.

Cross-reactivity was observed with two organisms. Off-panel *Candida* (*Yarrowia*) *deformans* was erroneously identified by the LIAISON PLEX BCY Assay as on-panel *Candida lipolytica* when tested at high concentrations, and the assay misidentified off-panel *Candida pseudohaemulonii* as *Candida haemulonii*/*C. duobushaemulonii*. Both of these cross-reactions were predicted by *in silico* analyses of the assay’s oligonucleotide sequences. *Candida* (*Yarrowia*) *deformans* is phenotypically similar to *Candida lipolytica* (also known as *Yarrowia lipolytica*), but the *Candida* (*Yarrowia*) *deformans* sp. is not a known mammalian pathogen. *Candida lipolytica* is an emergent and rare opportunistic pathogen that has been occasionally identified in eye, foodborne, and intravascular catheter-related infections (17). *Candida pseudohaemulonii*, *C. haemulonii*, and *C. duobushaemulonii* are all members of the *Metschnikowiaceae* clade of pathogenic yeasts closely related to *C. auris* (18). These fungal pathogens are all rare opportunists of tropical American origin, noted for broad intrinsic antifungal resistance (including against fluconazole and amphotericin B) and a high mortality rate. Bloodborne infection by off-panel *C. pseudohaemulonii* currently occurs much less frequently than infection by other related on-panel members of the *C. haemulonii* complex, but is probably clinically equivalent (18). Importantly, the BCY nucleic acid assay reliably differentiated *C. auris* from *C. haemulonii* complex members, a misidentification that commonly occurs when relying on culture methods alone for characterization (19). The BCY Assay consistently detected multiple strains of all on-panel fungal targets, and test performance was unaffected by the presence of various bacteria or off-panel fungi. No interference was observed in the simultaneous detection of combinations of on-panel fungi at varying concentrations, suggesting good BCY Assay performance in identifying polyfungal bloodstream infections.

Assay performance was unaffected by the inclusion of high concentrations of all endogenous and nearly all exogenous chemical additives that were tested (Table S4). Only fluconazole at 25 mg/L caused interference with *C. albicans* identification, resulting in a false-negative call. While this concentration approximates the median serum levels observed with high-dosage fluconazole therapy (e.g., 34‒35 µg/mL at 600‒800 mg/day dosing), it is unlikely that these drug levels will be encountered in diluted blood culture broth tested by the BCY Assay (20). No interference was observed by fluconazole at lower concentrations (e.g., 8.3 mg/L; Table S4). No interference occurred for any of the 16 on-panel fungal pathogens by other common antifungal agents (e.g., amphotericin B and caspofungin) or antibacterial drugs tested (Table S4), suggesting good assay performance with blood culture broth samples derived from patients who may be receiving antimicrobial therapy.

The outstanding clinical performance of the LIAISON PLEX BCY Assay was evidenced by achieving 100% sensitivity/PPA and ≥97.6% specificity/NPA for accurate identification of all target organisms in prospective, pre-selected, and contrived specimens. Three of the five false positive results from the prospective and pre-selected sample set (1/1 *C*. *albicans*, 1/1 *C*. *parapsilosis*, and 1/3 *C*. *tropicalis*) were confirmed as positive by the BCID2 molecular assay. Curiously, in the contrived sample set, none of the five false positive results (1/1 *C*. *famata*, 1/1 *C*. *parapsilosis*, and 3/3 *C*. *tropicalis*) could be confirmed by another method. The *C. famata* sample was not further tested, but BCID2 was used for additional testing of the *C. parapsilosis* and the three *C. tropicalis* samples. *C. tropicalis* had the lowest NPA overall at 95.2% (60/63) for the prospective sample set and 99.6% (775/778) for the contrived sample set. Of the total six false positive results, one was confirmed positive by the BCID2 assay, but the remaining five could not be confirmed as positive by another method. Assay performance was unaffected by the blood culture instrument or media type used and included BACT/ALERT VIRTUO, BD BACTEC™, and BACT/ALERT 3D blood culture systems for the prospective and pre-selected samples. While most post-culture fungal testing seeks to characterize a single suspected pathogen, the multiplex BCY Assay simultaneously analyzes samples for the presence of up to 16 known fungal pathogens, 8 of which are included in the WHO priority pathogen listing (8). Furthermore, the comprehensive fungal pathogen coverage of the LIAISON PLEX BCY Assay was confirmed in the prospective sample set where only one of 69 positive blood cultures contained a fungal pathogen (*Candida orthopsilosis*) that is not included in the panel targets.

The specific fungal pathogens responsible for bloodstream infection exhibit both geographic and temporal variance (2, 4, 21). For example, *C. albicans* was the causative pathogen for 70–80% of invasive candidiasis infections globally in the 1980s–1990s (22), but this proportion has now decreased to 40–60% in most areas (23). Also, susceptibility to infection by certain *Candida* spp. varies according to patient medical conditions and antimicrobial regimens; e.g., *C. glabrata* is more commonly isolated in patients with cancer, and *C. krusei* tends to affect patients with hematologic malignancy who receive prophylactic fluconazole (24). In our 69 prospective patient samples collected in the US, *C. glabrata* was the most frequently identified species (36%), followed by *C. albicans* (25%), and *C. parapsilosis* (16%). *Cryptococcus neoformans*/*C. gattii* was not identified in this cohort. The LIAISON PLEX BCY Assay offers clinicians a tailored diagnostic tool whereby fungal testing can be specified to evaluate only the pathogens most likely to be encountered, according to the Gram stain result.

As an adjunctive qualitative test, this assay is meant to provide rapid confirmation and clarification of positive continuous blood culture findings and not serve as a standalone diagnostic tool. While accurate identification was demonstrated for 5 tested sequence variants of each of the 16 assay targets, there still remains a risk of false-negative findings with other untested fungal sequence variants. A negative finding for all 16 BCY Assay targets does not preclude the possibility of infection by a fungus included in the test panel or of co-infection with other viruses and bacteria. It is possible that BCY Assay results may be affected by concurrent antimicrobial therapy or that levels of fungi in specimens are below the test’s limit of detection. Last, although this assay was shown to be compatible with pediatric culture bottles, it has not been validated for performance in pediatric patients, so test suitability for young patients is left to physician discretion. Strengths of this study included the multicenter prospective design and comprehensive performance assessment.

Other rapid syndromic molecular panels are also available for the qualitative detection and differentiation of fungal pathogens in blood cultures. The Roche cobas (GenMark Dx) ePlex Blood Culture Identification Fungal Pathogen Panel (BCID-FP) includes 15 fungal targets. As compared to the LIAISON PLEX BCY Assay, BCID-FP does not include *C. haemulonii*/*C. duobushaemulonii* or *C. lipolytica*, but it differentiates *C. neoformans* and *C. gattii* and includes *Fusarium* and *Rhodotorula*, which are not present in LIAISON PLEX BCY. As reported by Zhang et al., the BCID-FP panel demonstrated similar high sensitivity and specificity for detecting fungal pathogens in positive blood cultures, with sensitivities ranging from 97.1% to 100.0% and specificities from 99.8% to 100.0% (25). BCID-FP has a similarly fast workflow with 2 minute hands-on time and 1.5 h time-to-result (25, 26), as compared to a 1 minute hands-on time and 2 h to result for LIAISON PLEX BCY. The bioMérieux BIOFIRE BCID2 Panel, which was used for confirmatory testing in this study, includes 43 targets in a single panel for the detection and differentiation of bacteria, yeast, and antimicrobial resistance genes from positive blood cultures. The panel includes seven yeast species that correspond to targets present in LIAISON PLEX BCY (*C. albicans*, *C. auris*, *C. glabrata*, *C. krusei*, *C. parapsilosis*, *C. tropicalis*, and *C. neoformans*/*C. gattii*). In the multicenter study reported by Rhoads et al., BCID2 had sensitivities of 100% for five of the seven yeast targets that were present in the 1,074 samples that were included in the final analysis (27). Specificities ranged from 99.9% to 100.0%. The BCID2 assay has a time-to-result of approximately 1 h and a hands-on time of ≤10 minutes (27, 28).

In conclusion, this study demonstrated the excellent sensitivity and specificity of the multiplex LIAISON PLEX BCY nucleic acid diagnostic assay for simultaneously evaluating 16 known fungal pathogens in positive blood culture specimens. Although culture techniques remain the gold standard approach for evaluating bloodstream fungal infection, the BCY Assay provides excellent performance in quickly assessing the presence of multiple known pathogenic fungi in positive blood cultures.

## Data Availability

The data supporting the findings of this study are available from the corresponding author upon reasonable request.

## SUPPLEMENTAL MATERIAL

Supplemental tables jcm.00362-25-s0001.docx Tables S1 to S8.

ASM does not own the copyrights to Supplemental Material that may be linked to, or accessed through, an article. The authors have granted ASM a non-exclusive, world-wide license to publish the Supplemental Material files. Please contact the corresponding author directly for reuse.

## References

[1] Denning DW. 2024. Global incidence and mortality of severe fungal disease. Lancet Infect Dis 24:e428–e438. doi:10.1016/S1473-3099(23)00692-8 38224705

[2] Bays DJ, Jenkins EN, Lyman M, Chiller T, Strong N, Ostrosky-Zeichner L, Hoenigl M, Pappas PG, Thompson Iii GR. 2024. Epidemiology of invasive candidiasis. Clin Epidemiol 16:549–566. doi:10.2147/CLEP.S459600 39219747 PMC11366240

[3] Bongomin F, Gago S, Oladele RO, Denning DW. 2017. Global and multi-national prevalence of fungal diseases-estimate precision. J Fungi (Basel) 3:57. doi:10.3390/jof3040057 29371573 PMC5753159

[4] Wolfgruber S, Sedik S, Klingspor L, Tortorano A, Gow NAR, Lagrou K, Gangneux JP, Maertens J, Meis JF, Lass-Flörl C, Arikan-Akdagli S, Cornely OA, Hoenigl M. 2024. Insights from three pan-European multicentre studies on invasive candida infections and outlook to ECMM Candida IV. Mycopathologia 189:70. doi:10.1007/s11046-024-00871-0 39088098 PMC11294264

[5] Benedict K, Jackson BR, Chiller T, Beer KD. 2019. Estimation of direct healthcare costs of fungal diseases in the United States. Clin Infect Dis 68:1791–1797. doi:10.1093/cid/ciy776 30204844 PMC6409199

[6] Barantsevich N, Barantsevich E. 2022. Diagnosis and treatment of invasive candidiasis. Antibiotics (Basel) 11:718. doi:10.3390/antibiotics11060718 35740125 PMC9219674

[7] Kim JS, Cha H, Bahn YS. 2024. Comprehensive overview of Candida auris: an emerging multidrug-resistant fungal pathogen. J Microbiol Biotechnol 34:1365–1375. doi:10.4014/jmb.2404.04040 38881183 PMC11294645

[8] World Health Organization. 2022. WHO fungal priority pathogens list to guide research, development and public health action. World Health Organization. Available from: https://www.who.int/publications/i/item/9789240060241

[9] Azim A, Ahmed A. 2024. Diagnosis and management of invasive fungal diseases in non-neutropenic ICU patients, with focus on candidiasis and aspergillosis: a comprehensive review. Front Cell Infect Microbiol 14:1256158. doi:10.3389/fcimb.2024.1256158 38505289 PMC10948617

[10] Clancy CJ, Nguyen MH. 2013. Finding the "missing 50%" of invasive candidiasis: how nonculture diagnostics will improve understanding of disease spectrum and transform patient care. Clin Infect Dis 56:1284–1292. doi:10.1093/cid/cit006 23315320

[11] Osset-Trénor P, Pascual-Ahuir A, Proft M. 2023. Fungal drug response and antimicrobial resistance. J Fungi (Basel) 9:565. doi:10.3390/jof9050565 37233275 PMC10219139

[12] Knoll MA, Ulmer H, Lass-Flörl C. 2021. Rapid antifungal susceptibility testing of yeasts and molds by MALDI-TOF MS: a systematic review and meta-analysis. J Fungi (Basel) 7:63. doi:10.3390/jof7010063 33477533 PMC7835946

[13] Jenks JD, White PL, Kidd SE, Goshia T, Fraley SI, Hoenigl M, Thompson GR 3rd. 2023. An update on current and novel molecular diagnostics for the diagnosis of invasive fungal infections. Expert Rev Mol Diagn 23:1135–1152. doi:10.1080/14737159.2023.2267977 37801397 PMC10842420

[14] Alves J, Alonso-Tarrés C, Rello J. 2022. How to identify invasive candidemia in ICU-A narrative review. Antibiotics (Basel) 11:1804. doi:10.3390/antibiotics11121804 36551461 PMC9774599

[15] United States Food and Drug Administration. 2024. K240627. LIAISON PLEX yeast blood culture assay. 510(k) substantial equivalence determination; decision summary. United States Food and Drug Administration. Available from: https://www.accessdata.fda.gov/scripts/cdrh/cfdocs/cfpmn/pmn.cfm?ID=K240627. Retrieved 25 Nov 2024.

[16] Diasorin. 2024. The LIAISON PLEX system. Available from: https://us.diasorin.com/en/molecular-diagnostics/tools/liaison-plex?f%5B0%5D=mdx_disease%3A249. Retrieved 25 Nov 2024.

[17] Yu J, Liu X, Guo D, Yang W, Chen X, Zou G, Wang T, Pang S, Zhang G, Dong J, Xu Y, Zhao Y. 2024. Antifungal susceptibility profile and local epidemiological cut-off values of Yarrowia (Candida) lipolytica: an emergent and rare opportunistic yeast. Microbiol Spectr 12:e0320323. doi:10.1128/spectrum.03203-23 38084981 PMC10783140

[18] Françoise U, Desnos-Ollivier M, Le Govic Y, Sitbon K, Valentino R, Peugny S, Chouaki T, Mazars E, Paugam A, Nicolas M, Desbois-Nogard N, Lortholary O, French Mycoses Study Group. 2023. Candida haemulonii complex, an emerging threat from tropical regions? PLoS Negl Trop Dis 17:e0011453. doi:10.1371/journal.pntd.0011453 37523406 PMC10437918

[19] Kathuria S, Singh PK, Sharma C, Prakash A, Masih A, Kumar A, Meis JF, Chowdhary A. 2015. Multidrug-resistant Candida auris misidentified as Candida haemulonii: characterization by matrix-assisted laser desorption ionization-time of flight mass spectrometry and DNA sequencing and its antifungal susceptibility profile variability by Vitek 2, CLSI broth microdilution, and Etest method. J Clin Microbiol 53:1823–1830. doi:10.1128/JCM.00367-15 25809970 PMC4432077

[20] Schiave LA, Nascimento E, Vilar FC, de Haes TM, Takayanagui OM, Gaitani C de, Martinez R. 2018. Fluconazole levels in serum and cerebrospinal fluid according to daily dosage in patients with cryptococcosis and other fungal infections. Braz J Infect Dis 22:11–15. doi:10.1016/j.bjid.2017.10.003 29144957 PMC9425654

[21] Pappas PG, Lionakis MS, Arendrup MC, Ostrosky-Zeichner L, Kullberg BJ. 2018. Invasive candidiasis. Nat Rev Dis Primers 4:18026. doi:10.1038/nrdp.2018.26 29749387

[22] Wenzel RP. 1995. Nosocomial candidemia: risk factors and attributable mortality. Clin Infect Dis 20:1531–1534. doi:10.1093/clinids/20.6.1531 7548504

[23] Hoenigl M, Salmanton-García J, Egger M, Gangneux JP, Bicanic T, Arikan-Akdagli S, Alastruey-Izquierdo A, Klimko N, Barac A, Özenci V, et al.. 2023. Guideline adherence and survival of patients with candidaemia in Europe: results from the ECMM Candida III multinational European observational cohort study. Lancet Infect Dis 23:751–761. doi:10.2139/ssrn.4234174 37254300

[24] McCarty TP, White CM, Pappas PG. 2021. Candidemia and invasive candidiasis. Infect Dis Clin North Am 35:389–413. doi:10.1016/j.idc.2021.03.007 34016283

[25] Zhang SX, Carroll KC, Lewis S, Totten M, Mead P, Samuel L, Steed LL, Nolte FS, Thornberg A, Reid JL, Whitfield NN, Babady NE. 2020. Multicenter evaluation of a PCR-based digital microfluidics and electrochemical detection system for the rapid identification of 15 fungal pathogens directly from positive blood cultures. J Clin Microbiol 58:e02096-19. doi:10.1128/JCM.02096-19 32075904 PMC7180249

[26] Mizusawa M, Carroll KC. 2023. Updates on the profile of GenMark’s ePlex blood culture identification fungal pathogen panel. Expert Rev Mol Diagn 23:475–484. doi:10.1080/14737159.2023.2215929 37198873

[27] Rhoads DD, Pournaras S, Leber A, Balada-Llasat JM, Harrington A, Sambri V, She R, Berry GJ, Daly J, Good C, Tarpatzi A, Everhart K, Henry T, McKinley K, Zannoli S, Pak P, Zhang F, Barr R, Holmberg K, Kensinger B, Lu DY. 2023. Multicenter evaluation of the BIOFIRE blood culture identification 2 panel for detection of bacteria, yeasts, and antimicrobial resistance genes in positive blood culture samples. J Clin Microbiol 61:e0189122. doi:10.1128/jcm.01891-22 37227281 PMC10281132

[28] Smith RD, Zhan M, Zhang S, Leekha S, Harris A, Doi Y, Evans S, Kristie Johnson J, Ernst RK. 2024. Comparison of three rapid diagnostic tests for bloodstream infections using Benefit-risk Evaluation Framework (BED-FRAME). J Clin Microbiol 62:e0109623. doi:10.1128/jcm.01096-23 38054730 PMC10793330

