# Supplementary material for: Performance of the LIAISON PLEX yeast blood culture assay for identifying 16 invasive fungal pathogens in blood cultures
Source: J Clin Microbiol. 2025 Jun 2;63(7):e00362-25. doi: 10.1128/jcm.00362-25 (PMC12239718; doi:10.1128/jcm.00362-25)
Supplement: Supplemental tables — Tables S1 to S8. [file jcm.00362-25-s0001.docx]

**SUPPLEMENTAL TABLES**

**Supplemental Table S1.** Target detectability by the LIAISON PLEX BCY Assay

| **LIAISON PLEX^®^ BCY Assay Result** | **Growth** | **Average Concentration (CFU/mL)** | **Target Positivity** |
| --- | --- | --- | --- |
| *Candida albicans* | Ring Positive | 4.37E+05 | 100% (9/9) |
|  | Ring Positive + 8h | 8.04E+06 | 100% (9/9) |
| *Candida auris* | Ring Positive | 1.78E+07 | 100% (9/9) |
|  | Ring Positive + 8h | 4.77E+07 | 100% (9/9) |
| *Candida dubliniensis* | Ring Positive | 2.22E+07 | 100% (9/9) |
|  | Ring Positive + 8h | 4.50E+07 | 100% (9/9) |
| *Candida famata* | Ring Positive | 5.13E+06 | 100% (9/9) |
|  | Ring Positive + 8h | 1.20E+07 | 100% (9/9) |
| *Candida glabrata* | Ring Positive | 5.97E+06 | 100% (9/9) |
|  | Ring Positive + 8h | 1.47E+08 | 100% (9/9) |
| *Candida guilliermondii* | Ring Positive | 2.46E+07 | 100% (9/9) |
|  | Ring Positive + 8h | 3.60E+07 | 100% (9/9) |
| *Candida haemulonii* | Ring Positive | 9.93E+06 | 100% (9/9) |
|  | Ring Positive + 8h | 3.67E+07 | 100% (9/9) |
| *Candida kefyr* | Ring Positive | 8.83E+06 | 100% (9/9) |
|  | Ring Positive + 8h | 2.19E+07 | 100% (9/9) |
| *Candida krusei* | Ring Positive | 1.66E+07 | 100% (9/9) |
|  | Ring Positive + 8h | 3.90E+07 | 100% (9/9) |
| *Candida lipolytica* | Ring Positive | 2.45E+06 | 100% (9/9) |
|  | Ring Positive + 8h | 4.10E+06 | 100% (9/9) |
| *Candida lusitaniae* | Ring Positive | 3.61E+07 | 100% (9/9) |
|  | Ring Positive + 8h | 1.29E+08 | 100% (9/9) |
| *Candida parapsilosis* | Ring Positive | 1.64E+07 | 100% (9/9) |
|  | Ring Positive + 8h | 4.50E+07 | 100% (9/9) |
| *Candida tropicalis* | Ring Positive | 1.29E+07 | 100% (9/9) |
|  | Ring Positive + 8h | 3.40E+07 | 100% (9/9) |
| *Cryptococcus neoformans* | Ring Positive | 2.44E+06 | 100% (9/9) |
|  | Ring Positive + 8h | 4.97E+06 | 100% (9/9) |
| Negative Blood Matrix | N/A | NA | 0% |

**Supplemental Table S2.** List of common commercial culture bottles/media that demonstrated compatibility with the LIAISON PLEX BCY Assay*^a^*

| **Bottle Manufacturer** | **Bottle Type** | **Bottle Catalog Number** |
| --- | --- | --- |
| Thermo Scientific | VersaTrek Redox 1 | 7106-44 |
|  | VersaTrek Redox 2 | 7107-44 |
| BD | Bactec Plus Anaerobic/F | 442022 |
|  | Bactec Standard Anaerobic/F | 442024 |
|  | Bactec Lytic/10 Anaerobic/F | 442021 |
|  | Bactec Plus Aerobic/F | 442023 |
|  | Bactec Peds Plus | 442020 |
|  | Bactec Standard/10 Aerobic/F | 442027 |
| BioMérieux | BACT/Alert SA | 259789 |
|  | BACT/Alert FA Plus*^b^* | 410851 |
|  | BACT/Alert FN Plus | 410852 |
|  | BACT/Alert SN | 259790 |
|  | BACT/Alert PF Plus | 410853 |

*^a^*All tested bottle types demonstrated 100% detection of six fungal pathogens (*C. albicans, C. tropicalis, C. neoformans, C. glabrata, C. guilliermondii,* and *C. kefyr*), with 0% false detection of 5 bacterial strains (*A. baumannii, E. coli, K. pneumoniae, P. aeruginosa,* and *S. aureus*) or negative blood cultures.

*^b^*The BioMérieux BACT/Alert FA Plus was used during analytical testing of this assay and is also considered compatible. It has been included in the list of compatible blood culture bottles.

**Supplemental Table S3**. Off-panel bacteria and fungi evaluated for cross-reactivity with the LIAISON PLEX BCY Assay.

| **Bacteria** | **Source & Source ID** | **Concentration Tested (CFU/mL)** |
| --- | --- | --- |
| *Acinetobacter baumannii* | IHMA 128307 | 1.0E+08 |
| *Acinetobacter Iwoffii* | ATCC 15309 | 1.0E+08 |
| *Aerococcus viridans* | ATCC 700406 | 1.0E+08 |
| *Bacillus cereus* | ATCC 14579 | 1.0E+08 |
| *Bacteroides fragilis* | ATCC 25285 | 1.0E+08 |
| *Bordetella pertussis* | ATCC 9797 | 1.0E+08 |
| *Cutibacterium (Propionibacterium) acnes* | ATCC 33179 | 1.0E+08 |
| *Citrobacter freundii* | ATCC 8090 | 1.0E+08 |
| *Clostridium perfringens* | ATCC 13124 | 1.0E+08 |
| *Corynebacterium striatum* | ATCC 43735 | 1.0E+08 |
| *Enterobacter aerogenes* | ATCC 35029 | 1.0E+08 |
| *Enterococcus avium* | ATCC 14025 | 1.0E+08 |
| *Enterobacter cloacae* | ATCC 35030 | 1.0E+08 |
| *Escherichia coli* | NCTC 13846 | 1.0E+08 |
| *Enterococcus faecalis* | ATCC 29212 | 1.0E+08 |
| *Enterococcus faecium* | ATCC 700221 | 1.0E+08 |
| *Eggerthella lenta* | ATCC 25559 | 1.0E+08 |
| *Klebsiella oxytoca* | ATCC 43165 | 1.0E+08 |
| *Klebsiella pneumoniae* | ATCC-BAA 2146 | 1.0E+08 |
| *Listeria monocytogenes* | ATCC 19115 | 1.0E+08 |
| *Lactobacillus rhamnosus* | ATCC 53103 | 1.0E+08 |
| *Micrococcus luteus* | ATCC 10054 | 8.9E+06 |
| *Morganella morganii* | ATCC 25830 | 1.0E+08 |
| *Pseudomonas aeruginosa* | IHMA 576602 | 1.0E+08 |
| *Prevotella denticola* | BEI Resources HM-1173 | 1.0E+08 |
| *Proteus mirabilis* | ATCC 12453 | 1.0E+08 |
| *Streptococcus agalactiae* | ATCC 12401 | 1.0E+08 |
| *Streptococcus anginosus* | ATCC 33397 | 1.0E+08 |
| *Staphylococcus aureus* | ATCC 25923 | 1.0E+08 |
| *Salmonella enterica* | ATCC 9993 | 1.0E+08 |
| *Staphylococcus epidermidis* | ATCC 700567 | 1.0E+08 |
| *Staphylococcus hominis* | ATCC 27844 | 1.0E+08 |
| *Staphylococcus intermedius* | ATCC 29663 | 1.0E+08 |
| *Staphylococcus lugdunensis* | ATCC 43809 | 1.0E+08 |
| *Stenotrophomonas maltophilia* | ATCC 17666 | 8.9E+07 |
| *Serratia marcescens* | NCTC 13920 | 1.0E+08 |
| *Streptococcus mitis* | ATCC 15914 | 1.0E+08 |
| *Streptococcus pneumoniae* | ATCC 6315 | 1.0E+08 |
| *Streptococcus pyogenes* | ATCC 12964 | 1.0E+08 |
| *Staphylococcus saprophyticus* | ATCC 15305 | 1.0E+08 |
| **Fungi** | **Source & Source ID** | **Concentration Tested (CFU/mL)** |
| *Aspergillus fumigatus* | ATCC 1022 | 1.0E+07 |
| *Acremonium kiliense* | ATCC 20337 | 1.0E+07 |
| *Candida bracarensis* | JH 47 | 1.0E+07 |
| *Candida carpophila* | CBS-KNAW 5256 | 1.0E+07 |
| *Candida inconspicua* | NRRL Y-2029 | 1.0E+07 |
| *Candida intermedia* | ATCC 14439 | 1.0E+07 |
| *Candida metapsilosis* | NRRL Y-48470 | 1.0E+07 |
| *Candida nivariensis* | CBS-KNAW 9983 | 1.0E+07 |
| *Candida orthopsilosis* | ATCC 20504 | 1.0E+07 |
| *Candida pseudohaemulonii* | CBS-KNAW 10004 | 1.0E+02*^a^* |
| *Candida rugosa* | NRRL Y-95 | 1.0E+07 |
| *Candida sake* | NRRL Y-1622 | 1.0E+07 |
| *Candida solani* | NRRL Y-2224 | 1.0E+07 |
| *Candida utilis* | ATCC 9950 | 1.0E+07 |
| *Exophiala lecanii-corni* | ATCC 12734 | 1.0E+07 |
| *Filobasidium elegans* | NRRL Y-6486 | 1.0E+07 |
| *Filobasidium globisporum* | NRRL Y-17828 | 1.0E+07 |
| *Kluyveromyces lactis* | ATCC 24207 | 1.0E+07 |
| *Kodamaea ohmeri* | NRRL Y-1932 | 1.0E+07 |
| *Meyerozyma caribbica* | NRRL Y-27274 | 1.0E+07 |
| *Malassezia furfur* | ATCC 14521 | 1.0E+07 |
| *Malassezia globosa* | ATCC-MYA 4612 | 1.0E+07 |
| *Metschnikowia pulcherrima* | ATCC 22032 | 1.0E+07 |
| *Malassezia restricta* | ATCC-MYA 4611 | 1.0E+07 |
| *Malassezia sympodialis* | ATCC 96803 | 1.0E+07 |
| *Mucor velutinosus* | ATCC-MYA 4766 | 1.0E+07 |
| *Pichia fermentans* | NRRL Y-1619 | 1.0E+07 |
| *Pichia norvegensis* | NRRL YB-3904 | 1.0E+07 |
| *Rhodotorula mucilaginosa* | ATCC 66034 | 1.0E+07 |
| *Saccharomyces cerevisiae* | ATCC 18824 | 1.0E+07 |
| *Scedosporium prolificans* | ATCC 64913 | 1.0E+07 |
| *Sporidiobolus salmonicolor* | NRRL Y-5483 | 1.0E+07 |
| *Sporothrix schenckii* | ATCC 58251 | 1.0E+07 |
| *Trichosporon asteroides* | CBS-KNAW 6183 | 1.0E+07 |
| *Talaromyces marneffei* | ATCC 18224 | 1.45E+06 spores/mL |
| *Wickerhamomyces anomalus* | ATCC 10262 | 1.0E+07 |
| *Yarrowia deformans* | NRRL Y-63659 | 1.0E+04*^b^* |

*^a^Candida pseudohaemulonii* at a concentration greater than 1.0E+02 demonstrated cross-reactivity with *C. haemulonii/duobushhaemulonii*.

*^b^Yarrowia deformans* at a concentration greater than 1.0E+04 demonstrated cross-reactivity with *C. lipolytica*.

**Supplemental Table S4**. Effect of potentially interfering substances on the LIAISON PLEX BCY Assay

| **Endogenous Substances** | **Testing Concentration** |
| --- | --- |
| Unconjugated Bilirubin | 20 mg/dL |
| Conjugated Bilirubin | 20 mg/dL |
| Hemoglobin | 14 g/L |
| Intralipid/Triglycerides | 3000 mg/dL |
| γ-globulin | 6 g/dL |
| **Exogenous Substances** | **Testing Concentration** |
| Sodium Polyanethol Sulfonate | 0.25% w/v |
| Amoxicillin clavulanate | 3.5 µg/mL |
| Amphotericin B | 2 µg/mL |
| Caspofungin | 5 µg/mL |
| Ceftriaxone | 0.23 mg/mL |
| Ciprofloxacin | 3 mg/L |
| Fluconazole | 8.3 mg/L*^a^* |
| Flucytosine | 90 µg/mL |
| Gentamicin sulfate | 3 µg/mL |
| Heparin | 0.9 U/mL |
| Imipenem | 83 µg/mL |
| Tetracycline | 5 mg/L |
| Vancomycin | 30 mg/L |

*^a^*Fluconazole caused interference and resulted in a false-negative call for *C. albicans* when tested at a concentration of 25 mg/L.

**Supplemental Table S5.** Clinical performance of the LIAISON PLEX BCY Assay

| **Pathogen Target** | | **Sensitivity / PPA*^a^*** | | | **Specificity / NPA*^b^*** | | |
| --- | --- | --- | --- | --- | --- | --- | --- |
|  | | **TP / (TP+FN)** | **Sensitivity / PPA** | **95% CI** | **TN / (TN+FP)** | **Specificity / NPA** | **95% CI** |
| *Candida albicans* | Prospective | 17/17 | 100% | 81.6%-100% | 51/52*^c^* | 98.1% | 89.9%-99.7% |
|  | Pre-selected | 17/17 | 100% | 81.6%-100% | 46/46 | 100% | 92.3%-100% |
|  | **Combined** | **34/34** | **100%** | **89.8%-100%** | **97/98** | **99.0%** | **94.4%-99.8%** |
| *Candida auris* | Prospective | 4/4 | 100% | 51.0%-100% | 65/65 | 100% | 94.4%-100% |
|  | Pre-selected | 0/0 | NA | NA | 63/63 | 100% | 94.3%-100% |
|  | **Combined** | **4/4** | **100%** | **51.0%-100%** | **128/128** | **100%** | **97.1%-100%** |
| *Candida dubliniensis* | Prospective | 0/0 | NA | NA | 69/69 | 100% | 94.7%-100% |
|  | Pre-selected | 0/0 | NA | NA | 63/63 | 100% | 94.3%-100% |
|  | **Combined** | **0/0** | **NA** | **NA** | **132/132** | **100%** | **97.2%-100%** |
| *Candida famata* | Prospective | 0/0 | NA | NA | 69/69 | 100% | 94.7%-100% |
|  | Pre-selected | 0/0 | NA | NA | 63/63 | 100% | 94.3%-100% |
|  | **Combined** | **0/0** | **NA** | **NA** | **132/132** | **100%** | **97.2%-100%** |
| *Candida glabrata* | Prospective | 25/25 | 100% | 86.7%-100% | 44/44 | 100% | 92.0%-100% |
|  | Pre-selected | 21/21 | 100% | 84.5%-100% | 42/42 | 100% | 91.6%-100% |
|  | **Combined** | **46/46** | **100%** | **92.3%-100%** | **86/86** | **100%** | **95.7%-100%** |
| *Candida guilliermondii* | Prospective | 0/0 | NA | NA | 69/69 | 100% | 94.7%-100% |
|  | Pre-selected | 0/0 | NA | NA | 63/63 | 100% | 94.3%-100% |
|  | **Combined** | **0/0** | **NA** | **NA** | **132/132** | **100%** | **97.2%-100%** |
| *Candida haemulonii /C. duobushaemulonii* | Prospective | 0/0 | NA | NA | 69/69 | 100% | 94.7%-100% |
|  | Pre-selected | 0/0 | NA | NA | 63/63 | 100% | 94.3%-100% |
|  | **Combined** | **0/0** | **NA** | **NA** | **132/132** | **100%** | **97.2%-100%** |
| *Candida kefyr* | Prospective | 1/1 | 100% | 20.7%-100% | 68/68 | 100% | 94.7%-100% |
|  | Pre-selected | 0/0 | NA | NA | 63/63 | 100% | 94.3%-100% |
|  | **Combined** | **1/1** | **100%** | **20.7%-100%** | **131/131** | **100%** | **97.2%-100%** |
| *Candida krusei* | Prospective | 3/3 | 100% | 43.9%-100% | 66/66 | 100% | 94.5%-100% |
|  | Pre-selected | 1/1 | 100% | 20.7%-100% | 62/62 | 100% | 94.2%-100% |
|  | **Combined** | **4/4** | **100%** | **51.0%-100%** | **128/128** | **100%** | **97.1%-100%** |
| *Candida lipolytica* | Prospective | 0/0 | NA | NA | 69/69 | 100% | 94.7%-100% |
|  | Pre-selected | 0/0 | NA | NA | 63/63 | 100% | 94.3%-100% |
|  | **Combined** | **0/0** | **NA** | **NA** | **132/132** | **100%** | **97.2%-100%** |
| *Candida lusitaniae* | Prospective | 2/2 | 100% | 34.2%-100% | 67/67 | 100% | 94.6%-100% |
|  | Pre-selected | 0/0 | NA | NA | 63/63 | 100% | 94.3%-100% |
|  | **Combined** | **2/2** | **100%** | **34.2%-100%** | **130/130** | **100%** | **97.1%-100%** |
| *Candida parapsilosis* | Prospective | 11/11 | 100% | 74.1%-100% | 57/58*^d^* | 98.3% | 90.9%-99.7% |
|  | Pre-selected | 6/6 | 100% | 61.0%-100% | 57/57 | 100% | 93.7%-100% |
|  | **Combined** | **17/17** | **100%** | **81.6%-100%** | **114/115** | **99.1%** | **95.2%-99.8%** |
| *Candida tropicalis* | Prospective | 6/6 | 100% | 61.0%-100% | 60/63*^e^* | 95.2% | 86.9%-98.4% |
|  | Pre-selected | 0/0 | NA | NA | 63/63 | 100% | 94.3%-100% |
|  | **Combined** | **6/6** | **100%** | **61.0%-100%** | **123/126** | **97.6%** | **93.2%-99.2%** |
| *Cryptococcus neoformans/Cryptococcus gattii* | Prospective | 0/0 | NA | NA | 69/69 | 100% | 94.7%-100% |
|  | Pre-selected | 5/5 | 100% | 56.6%-100% | 58/58 | 100% | 93.8%-100% |
|  | **Combined** | **5/5** | **100%** | **56.6%-100%** | **127/127** | **100%** | **97.1%-100%** |

*^a^*Sensitivity is designated for prospective sample testing, while PPA is designated for pre-selected sample testing.

*^b^*Specificity is designated for prospective sample testing, while NPA is designated for pre-selected sample testing.

*^c^*The one *Candida albicans* False Positive was positive by the BCID2 molecular assay.

*^d^*The one *Candida parapsilosis* False Positive was positive by the BCID2 molecular assay.

*^e^*One of three *Candida tropicalis* False Positives was positive by the BCID2 molecular assay.

**Supplemental Table S6.** Limit-of-Detection for LIAISON PLEX Assay target fungal organisms*^a^*

| **On-Panel Fungal Target** | **Source & ID** | **Concentration (CFU/mL)** | **Target Positivity** |
| --- | --- | --- | --- |
| *Candida albicans* | ATCC 10231 | 3.14E+04 | 100% |
|  | ATCC 14053 | 2.83E+05 | 100% |
| *Candida auris* | CBS-KNAW 10913 | 3.50E+03 | 95% |
|  | CBS-KNAW 12766 | 3.50E+03 | 95% |
| *Candida dubliniensis* | ATCC-MYA 578 | 3.16E+03 | 100% |
|  | ATCC-MYA 577 | 3.16E+03 | 95% |
| *Candida famata* | ATCC 20278 | 7.77E+02 | 95% |
|  | ATCC 60229 | 6.99E+03 | 95% |
| *Candida glabrata* | ATCC 15545 | 9.99E+03 | 100% |
|  | ATCC 15126 | 1.00E+04 | 95% |
| *Candida guilliermondii* | ATCC 22017 | 3.33E+03 | 95% |
|  | ATCC 34134 | 1.00E+04 | 100% |
| *Candida haemulonii* | CBS-KNAW 7375 | 3.51E+03 | 95% |
| *Candida duobushaemulonii* | CBS-KNAW 7798 | 3.51E+03 | 100% |
| *Candida kefyr* | ATCC 8553 | 3.33E+03 | 95% |
|  | ATCC 4135 | 3.33E+03 | 100% |
| *Candida krusei* | ATCC 6258 | 9.83E+03 | 100% |
|  | ATCC 28870 | 2.95E+04 | 100% |
| *Candida lipolytica* | ATCC 20460 | 3.18E+04 | 100% |
|  | ATCC 20177 | 3.19E+04 | 100% |
| *Candida lusitaniae* | ATCC 42720 | 1.11E+04 | 100% |
|  | ATCC 34449 | 3.33E+04 | 100% |
| *Candida parapsilosis* | ATCC 28474 | 2.85E+04 | 100% |
|  | ATCC 28475 | 9.50E+03 | 100% |
| *Candida tropicalis* | ATCC 13803 | 3.51E+03 | 100% |
|  | ATCC 201380 | 3.51E+03 | 95% |
| *Cryptococcus neoformans* | ATCC 208821 | 9.01E+04 | 100% |
| *Cryptococcus gattii* | ATCC-MYA 4871 | 9.00E+04 | 95% |

*^a^*Limit of detection is defined as the lowest concentration where ≥95% (≥19/20) of samples tested positive.

**Supplemental Table S7.** Lack of inhibition of LIAISON PLEX BCY Assay target detection by high concentrations of potential bacterial interferents

| **Off-Panel High Concentration Interfering Bacterium** | **On-Panel Low Concentration Fungal Target** | **Target Positivity** |
| --- | --- | --- |
| *Escherichia coli*  (1.0 x 10^8^ CFU/mL) | *Candida albicans*  (4.4 x 10^5^ CFU/mL) | 100% (3/3) |
|  | *Candida glabrata*  (3.8 x 10^6^ CFU/mL) | 100% (3/3) |
|  | *Candida parapsilosis*  (1.5 x 10^7^ CFU/mL) | 100% (3/3) |
| *Staphylococcus aureus*  (1.0 x 10^8^ CFU/mL) | *Candida albicans*  (4.4 x 10^5^ CFU/mL) | 100% (3/3) |
|  | *Candida glabrata*  (3.8 x 10^6^ CFU/mL) | 100% (3/3) |
|  | *Candida parapsilosis*  (1.5 x 10^7^ CFU/mL) | 100% (3/3) |
| *Klebsiella pneumoniae*  (1.0 x 10^8^ CFU/mL) | *Candida albicans*  (4.4 x 10^5^ CFU/mL) | 100% (3/3) |
|  | *Candida glabrata*  (3.8 x 10^6^ CFU/mL) | 100% (3/3) |
|  | *Candida parapsilosis*  (1.5 x 10^7^ CFU/mL) | 100% (3/3) |
| *Pseudomonas aeruginosa*  (1.0 x 10^8^ CFU/mL) | *Candida albicans*  (4.4 x 10^5^ CFU/mL) | 100% (3/3) |
|  | *Candida glabrata*  (3.8 x 10^6^ CFU/mL) | 100% (3/3) |
|  | *Candida parapsilosis*  (1.5 x 10^7^ CFU/mL) | 100% (3/3) |
| *Enterococcus faecalis*  (1.0 x 10^8^ CFU/mL) | *Candida albicans*  (4.4 x 10^5^ CFU/mL) | 100% (3/3) |
|  | *Candida glabrata*  (3.8 x 10^6^ CFU/mL) | 100% (3/3) |
|  | *Candida parapsilosis*  (1.5 x 10^7^ CFU/mL) | 100% (3/3) |
| *Staphylococcus epidermidis*  (1.0 x 10^8^ CFU/mL) | *Candida albicans*  (4.4 x 10^5^ CFU/mL) | 100% (3/3) |
|  | *Candida glabrata*  (3.8 x 10^6^ CFU/mL) | 100% (3/3) |
|  | *Candida parapsilosis*  (1.5 x 10^7^ CFU/mL) | 100% (3/3) |
| *Enterococcus faecium*  (1.0 x 10^8^ CFU/mL) | *Candida albicans*  (4.4 x 10^5^ CFU/mL) | 100% (3/3) |
|  | *Candida glabrata*  (3.8 x 10^6^ CFU/mL) | 100% (3/3) |
|  | *Candida parapsilosis*  (1.5 x 10^7^ CFU/mL) | 100% (3/3) |
| *Acinetobacter baumannii*  (1.0 x 10^8^ CFU/mL) | *Candida albicans*  (4.4 x 10^5^ CFU/mL) | 100% (3/3) |
|  | *Candida glabrata*  (3.8 x 10^6^ CFU/mL) | 100% (3/3) |
|  | *Candida parapsilosis*  (1.5 x 10^7^ CFU/mL) | 100% (3/3) |
| *Streptococcus pneumoniae*  (1.0 x 10^8^ CFU/mL) | *Candida albicans*  (4.4 x 10^5^ CFU/mL) | 100% (3/3) |
|  | *Candida glabrata*  (3.8 x 10^6^ CFU/mL) | 100% (3/3) |
|  | *Candida parapsilosis*  (1.5 x 10^7^ CFU/mL) | 100% (3/3) |

**Supplemental Table S8.** Multi-site reproducibility of the LIAISON PLEX BCY Assay.

| **Organism** | **Target Type** | **Agreement with Expected Results** | | | | **95% C.I.** | |
| --- | --- | --- | --- | --- | --- | --- | --- |
|  |  | **Site 1** | **Site 2** | **Site 3** | **Overall** | **Lower** | **Upper** |
| *C. albicans* | Ring Positive | 100% (30/30) | 96.7% (29/30) | 100% (30/30) | 98.9% (89/90) | 94.0% | 99.8% |
|  | Ring Positive +8 Hours | 100% (30/30) | 100% (30/30) | 100% (30/30) | 100% (90/90) | 95.9% | 100% |
|  | Negative Blood Matrix | 100%  (30/30) | 100%  (30/30) | 100%  (30/30) | 100% (90/90) | 95.9% | 100% |
| *C. tropicalis* | Ring Positive | 100% (30/30) | 100% (30/30) | 100% (30/30) | 100% (90/90) | 95.9% | 100% |
|  | Ring Positive +8 Hours | 100% (30/30) | 100% (30/30) | 100% (30/30) | 100% (90/90) | 95.9% | 100% |
|  | Negative Blood Matrix | 100%  (30/30) | 100%  (30/30) | 100%  (30/30) | 100% (90/90) | 95.9% | 100% |
| *C. neoformans* | Ring Positive | 100% (30/30) | 100% (30/30) | 100% (30/30) | 100% (90/90) | 95.9% | 100% |
|  | Ring Positive +8 Hours | 100% (30/30) | 100% (30/30) | 100% (30/30) | 100% (90/90) | 95.9% | 100% |
|  | Negative Blood Matrix | 100%  (30/30) | 100%  (30/30) | 100%  (30/30) | 100% (90/90) | 95.9% | 100% |
| *E. coli* | Off-panel Negative | 100% (30/30) | 100% (30/30) | 100% (30/30) | 100% (90/90) | 95.9% | 100% |
